# Patients’ perceived needs of osteoarthritis health information: A systematic scoping review

**DOI:** 10.1371/journal.pone.0195489

**Published:** 2018-04-16

**Authors:** Louisa Chou, Lisa Ellis, Michelle Papandony, K. L. Maheeka D. Seneviwickrama, Flavia M. Cicuttini, Kaye Sullivan, Andrew J. Teichtahl, Yuanyuan Wang, Andrew M. Briggs, Anita E. Wluka

**Affiliations:** 1 Department of Epidemiology and Preventative Medicine, School of Public Health and Preventative Medicine, Monash University, Melbourne, Victoria, Australia; 2 Monash University Library, Monash University, Melbourne, Victoria, Australia; 3 Baker IDI Heart and Diabetes Institute, Melbourne, Victoria, Australia; 4 School of Physiotherapy and Exercise Science, Curtin University, Perth, Western Australia, Australia; 5 Move: muscle, bone & joint health, Melbourne, Victoria, Australia; Ohio State University, UNITED STATES

## Abstract

**Background:**

Optimal management of osteoarthritis requires active patient participation. Understanding patients’ perceived health information needs is important in order to optimize health service delivery and health outcomes in osteoarthritis. We aimed to review the existing literature regarding patients’ perceived health information needs for OA.

**Methods:**

A systematic scoping review was performed of publications in MEDLINE, EMBASE, CINAHL and PsycINFO (1990–2016). Descriptive data regarding study design and methodology were extracted and risk of bias assessed. Aggregates of patients’ perceived needs of osteoarthritis health information were categorized.

**Results:**

30 studies from 2876 were included: 16 qualitative, 11 quantitative and 3 mixed-methods studies. Three areas of perceived need emerged: (1) Need for clear communication: terms used were misunderstood or had unintended connotations. Patients wanted clear explanations. (2) Need for information from various sources: patients wanted accessible health professionals with specialist knowledge of arthritis. The Internet, whilst a source of information, was acknowledged to have dubious reliability. Print media, television, support groups, family and friends were utilised to fulfil diverse information needs. (3) Needs of information content: patients desired more information about diagnosis, prognosis, management and prevention.

**Conclusions:**

Patients desire more information regarding the diagnosis of osteoarthritis, its impact on daily life and its long-term prognosis. They want more information not only about pharmacological management options, but also non-pharmacological options to help them manage their symptoms. Also, patients wanted this information to be delivered in a clear manner from multiple sources of health information. To address these gaps, more effective communication strategies are required. The use of a variety of sources and modes of delivery may enable the provision of complementary material to provide information more successfully, resulting in better patient adherence to guidelines and improved health outcomes.

## Introduction

Osteoarthritis(OA), the most common type of arthritis, affects approximately one in ten adults[1]. The impact of OA is significant with 80% of those with knee OA reporting limited mobility and 25% reporting trouble with activities of daily living[2]. As OA has no cure, and its prevalence increases with age, it is predicted to be the fourth leading cause of disability by 2020[3], with considerable socioeconomic impact. OA is currently estimated to account for 1 to 2.5% of the gross national product of several countries, including the United Kingdom, France, Australia, Canada and United States of America[4].

To address the growing burden of OA, numerous guidelines for its management have been developed[5–7]. These recommend non-pharmacological interventions such as exercise, weight loss, assistive devices, the provision of effective and individualised information, as well as, pharmacological treatments including simple analgesics and intra-articular corticosteroid injections. Joint replacement surgery has also been recommended for suitable patients[6]. However, despite consensus between the multiple guidelines, clinical practice does not adequately reflect the recommendations: approximately one third of individuals with OA fail to receive recommended care[8],[9]. This is further compounded by a sizeable proportion of patients with OA not participating in recommended self-care strategies[10].

The uptake and adherence to clinical practice guidelines by clinicians and effective self-care strategies by patients is challenging and determined by a complex interplay between health care providers, the patients and resources provided within the health care system. The successful implementation of core OA guideline recommendations around education and self-management are largely dependent on patient engagement[6]. In order for patients to actively participate in their management, they require an understanding of their condition. The current EULAR guidelines for non-pharmacological management of hip and knee OA recommend the regular provision of individualised health information specifically addressing the nature of OA, its pathogenesis and its conseqences[11]. Despite this, previous studies have identified shortcomings in this with only 25% of patients with OA receiving disease-specific education[8,12]. This apparent gap in health information delivery is reflected in the poor health literacy regarding OA, with 30% of people with physician-diagnosed arthritis not being aware of the type of arthritis they have[13]. This is concerning as patients with low health literacy have been demonstrated to have worse outcomes and poorer access to health services[14]. Thus a mismatch between patients’ perceived health information needs and the information provided may contribute to poor uptake of guideline recommendations and less optimal health outcomes. Therefore, we aimed to review the existing literature regarding patients’ perceived health information needs relating to OA.

## Methods

We performed a systematic review of published data to identify what is known about patients’ perceived health information needs related to OA within a larger project examining the patient perceived needs relating to musculoskeletal health[15]. Given the breadth of the topic and to allow a comprehensive exploration of the patient perspective, a systematic scoping review was performed based on the framework proposed by Arksey and O’Malley[16]. Systematic scoping reviews are aimed at mapping key concepts, reviewing different types of evidence and identifying gaps in the current literature[17,18].

### Search strategy and study selection

A literature search was performed by electronically searching relevant databases (MEDLINE, EMBASE, CINAHL and PsycINFO) between January 1990-June 2016. A comprehensive search strategy was developed iteratively by a multidisciplinary team involving an academic librarian, patient input from one patient representative and four clinician researchers (Rheumatologists and Physiotherapist). The search strategy combined both MeSH terms and text words to capture information regarding patients' perceived health information needs related to OA (S1 Text. Search Strategy). Studies were not excluded based on study methods to capture the breadth of patients’ perspective on health information needs and OA.

Two investigators(LE, LC) assessed the titles and abstracts of all studies identified by the initial search for relevance. The initial screening was set to be open-ended to retain as many relevant studies as possible. Studies were included if they met the inclusion criteria: (1)studies had to concern patients older than 18 years, (2)studies had to report on patients’ perspectives regarding patient needs, expectations and requirements related to health information and (3)studies had to concern patients with OA of any joint. Studies were limited to the English language and full-text articles. Those that appeared to meet inclusion criteria were retrieved and the full text was assessed for relevance by two investigators (LE, LC). A manual search of the reference lists of the obtained studies was conducted to identify further studies for inclusion in the review.

### Methodological quality assessment

To assess the methodological quality of the included studies, two reviewers (LC and MP) independently assessed all of the included studies. For qualitative studies, the Critical Appraisal Skills Programme(CASP) tool was used[19]. Risk of bias tool was utilised to assess the external and internal validity of quantitative studies: low risk of bias was defined as scoring 8 or more “yes” answers, moderate risk of bias was defined as 6 to 7 “yes” answers and high risk of bias was defined as 5 or fewer “yes” answers[20]. The reviewers discussed and resolved disagreements through consensus. Any disagreements in scoring were reviewed by a third reviewer(AW).

### Data extraction and analysis

Two investigators(LE, LC) independently extracted the data from relevant studies using a standardised data extraction form developed for this scoping review. The following data were systematically extracted: (1)year of publication, (2)study population (patient age and gender, population source, population size and definition of OA), (3)primary study aim and (4)description of the study methods. Included studies were reviewed by two authors independently to identify aspects of health information for OA that patients had a preference for, expected, or were satisfied with using principles of meta-ethnography to synthesise qualitative data[21]. In the first stage, one author (LE) initially developed a framework of concepts and underlying themes, based on primary data in the studies and any pertinent points raised by the authors in the discussion. In the second stage, another author (LC) independently reviewed the studies and further developed the framework of themes and concepts. In the third stage two senior rheumatologists (FC, AW) with over 15 years of consultant experience, independently reviewed the framework of concepts and themes to ensure clinical meaningfulness and construct validity.

## Results

### Overview of included studies

**Fig 1:**
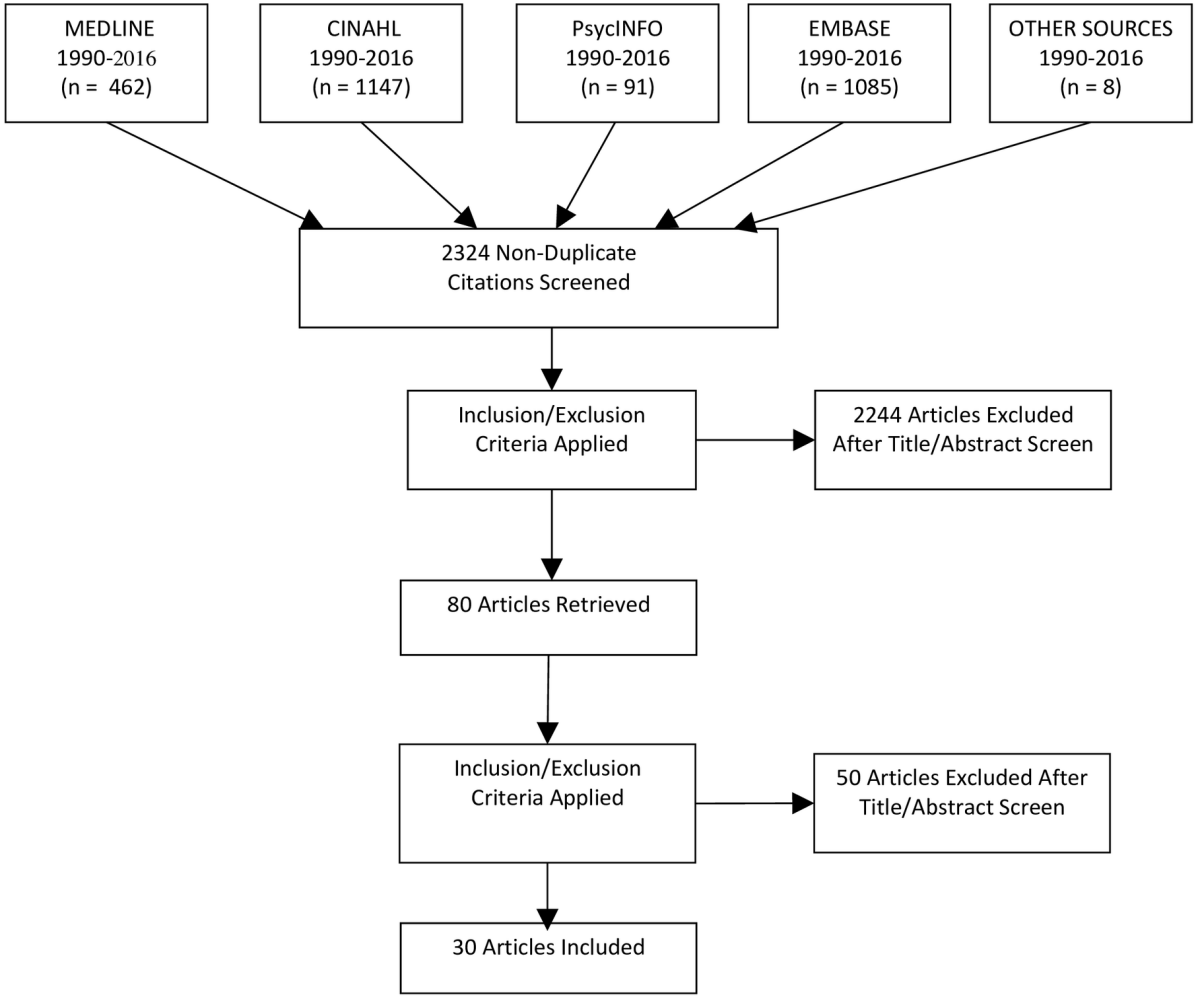
PRISMA diagram. *From*: Moher D, Liberati A, Tetzlaff J, Altman DG, The PRISMA Group (2009). *P*referred *R*eporting *I*terns for *S*ystematic Reviews and *M*eta-*A*nalyses: The PRISMA Statement. PLoS Med 6(7): e1000097. doi:10.1371/journal.pmed1000097. **For more information, visit** www.prisma-statement.org.

The search returned 2786 studies, of which 30 articles met inclusion criteria[22–51]. A PRISMA flow diagram detailing the study selection is shown in Fig 1. The descriptive characteristics of the included studies is shown in Table 1. The majority of studies were conducted in the United Kingdom[25,30,35,36,39,42,44,47,49,50], with the remainder from Europe[22,26,28,31,32,37,41,45,46,48], North America[27,33,43,51], South-East Asia[29,40], Australia[38], Middle-East[23] and an unknown source[24].

**Table 1:** Overview of studies.

Author and year	Diagnosis of knee OA	Number of Participants	Source of participants	Age and gender	Primary study aim	Study design
Al-Taiar [23] 2013 Kuwait	Not defined	39 participants	Women on the waiting list of public orthopaedic hospital for total knee arthroplasty	Mean age 62.5 100% female	This study aimed to explore the pain experience and mobility limitation as well as the patient’s decision-making process to undertake TKA among women with knee pain in the waiting list for surgery.	Qualitative Focus groups
Alami[22] 2011 France	Not defined	81 patients 29 care providers	Patients selected based on non-probability judgment sampling from Cochin Hospital (Paris).	29: 45–60 yrs 38: 61-80yrs 14: >80 yrs 73% female	To identify the views of patients and care providers regarding the management of knee OA and to reveal potential obstacles to improving health care strategies.	Qualitative Semi-structured interviews
Baird[24] 2003 Country unknown	Self-reported OA	5 participants	Purposive sampling of women >70 years attending a senior citizen centre	Mean age 78.2 100% female	To investigate “what is the meaning of self-caring for older women with physical functioning difficulties and OA?”	Qualitative In-depth interviews
Barker[25] 2014 UK	Self reported OA	39 participants recruited for focus groups and 6 individual interviews	Purposive sampling of patients from general public for focus groups; 6 members of public for interviews	Women >45 years of age 51% female	To explore the meanings and issues surrounding the use of existing medical terms for OA from the perspective of members of the public who have consulted healthcare practitioners for arthritis symptoms and from lay people who have not sought a consultation.	Qualitative Mixed methods combining focus groups and individual interviews
Baumann[26] 2007 France	Not defined	96 customers of 10 different pharmacies	First 10 customers of 10 pharmacies from 22 regions in France who visited pharmacies for their OA were recruited.	Mean age 65 81% female	To evaluate the expectations of OA patients in France and to consider how the information gathered may be used to improve the health care provision and patient-doctor relationship they received	Qualitative Focus group interviews
Bayliss[27] 2008 USA	Self reported diabetes, depression and OA	26 participants	26 patients randomly selected from 357 in a larger study within a not-for-profit Health Maintenance Organization (HMO)	Age range 65–84 years 50% female	To explore processes of care desired by elderly patients who have multimorbidities (OA, depression and Diabetes) that may present competing demands for patients and providers.	Qualitative Semi-structured interviews
Brembo[28] 2016 Norway	Not defined	13 participants	Purposive sample of patients with hip OA recruited from a GP practice and from the orthopaedic outpatient clinic at the local hospital	Age range 60–89 54% female	To investigate patients’ need for information and their personal emotional needs.	Qualitative Interviews
Chan[29] 2010 Hong Kong	ACR criteria of knee OA	20 participants	Convenience sampling of patients presenting to clinic	Mean age 57.05 (SD10.79) 65% female	To evaluate the influence of different pain patterns on the quality of life of patients with OA and to investigate their interpretation and coping strategies for the disease using patient interviews.	Qualitative Interviews
Clarke[30] 2014 UK	Radiographic OA (KL criteria), self reported OA and pain(74, 75)	216 patients (192 for questionnaire, 24 for interview)	474 invited to participate from the Arthritis Research UK Pain Centre (1) previous participants in a community-based study of knee OA (6), (2) Rheumatology and Orthopaedic clinics (Nottingham University Hospitals NHS Trust) (3) pre-operative assessment clinics (Sherwood Forest Hospitals NHS Trust)	Quantitative subject characteristics unknown. Qualitative study: Median age 62, 70% female	To examine the correspondence between qualitative and quantitative methods of coding experience of pain reported by participants with OA of the knee.	Quantitative Questionnaires Qualitative Semi-structured interviews
Cuperus[31] 2013 Netherlands	New episode of care attributed to symptomatic hip or knee OA (by GP)	17 participants	Sample of previous participants in project to implement a stepped care strategy for hip and knee OA in primary care.	Median age 67 (52–85), 71% female	To evaluate the introduction of the booklet “Care for Osteoarthritis” by (1) exploring how patients used the booklet and (2) identifying patient reported barriers and facilitators to use the booklet.	Qualitative Semi-structured interviews
Dragoi[32] 2013 Germany	ACR criteria	303 participants (130 had RA, 125 had PsA and 48 had hand OA).	Participants with RA, hand OA and PsA were asked to participate in the study	Hand OA patients mean age 64 (SD 7) and 83% female	To (i) develop and validate an Austrian-German version of the ENAT, (ii) to use the OENAT to explore educational needs of people with RA, PsA and hand OA, (iii) to search relationships between educational needs, gender, disease activity and functional ability	Quantitative Questionnaires
Fedutes[33] 2004 USA	Independent chart review, ICD codes	503 participants with 60% of participants having OA (28% response rate)	Cross-sectional study of 1800 patients (1079 OA patients and 661 RA patients and 60 with both RA and OA) of 7 physician community based university rheumatology practices	75% of respondents over the age of 56 67% female	To assess the interest of arthritis patients in an interactive, disease-specific arthritis management Website.	Quantitative Mail questionnaires
Gignac[34] 2006 Canada	ACR criteria	90 participants (53 with OA and 37 controls)	Purposive sampling, mild or moderate symptoms of knee OA from general practitioners, physical therapy clinics, the Arthritis Society Ontario Division, senior centres, fitness centres and advertisements in community newspapers.	Mean age 57 +/- 11 years 59% female	To compare the health experiences of middle and older age adults with moderate OA symptoms with experiences of individuals with no chronic health conditions.	Quantitative Focus groups and questionnaires
Grime[35] 2014 UK	Self-reported OA and participation in Research User Group	2 groups of lay advisors (11 in total)	A review of results from qualitative research into people’s experiences of living with chronic pain was used to structure group meetings	Age range 45-80yrs 75% female	To report on the experience of providing users with findings from qualitative research to increase awareness of their level of knowledge.	Qualitative Focus groups
Hill[36] 2011 UK	Clinical diagnosis of OA	17 patients in focus groups and interviews 29 patients in semi structured interviews	Purposive sampling of patients who were recruited from a GP and rheumatology department	Mean age 64.9 Focus group: 82% female Interviews: 86% female	To explore patients’ perceptions and experiences of the treatment and management of hand OA in older adults.	Qualitative Focus groups and semi structured interviews
Hofstede[37] 2016 Netherlands	Clinical diagnosis of hip or knee OA or previous total knee or hip arthroplasty	473 orthopaedic surgeons and 174 patients	Purposive sampling from academic and non-academic hospitals	Average age of patients 54 (S.D 7.7) and 72% female	To assess which barriers and facilitators are associated with the use and prescription of different non-surgical treatments before hip and knee OA in orthopaedic practice among patients and orthopaedic surgeons in the Netherlands	Quantitative Surveys
Ilic[38] 2005 Australia	Not defined	12 participants	12 patients recruited from “public advertisements”	Mean age 64 (SD 8.8) Gender unknown	To explore the feasibility of and user satisfaction with an Internet User’s Guide to assist patients in sourcing relevant, valid information about OA on the Internet.	Qualitative Focus groups
Jinks[39] 2007 UK	Knee pain (not necessarily knee OA)	Qualitative interviews were undertaken by 22 survey responder. 10 diaries	Patients were recruited from 3 general practices in North Staffordshire.	Age range 53-85yrs 45% female	To provide a model for knee pain and disability, describing felt need and expressed need.	Mixed methods Quantitative Questionnaires Qualitative In depth interviews, diaries
Kao[40] 2014 Taiwan	Clinical diagnosis and radiographic OA (Ahlback)	17 participants	Purposive sample of 23 potential participants were recruited via the orthopaedic clinics of two medical centres.	Mean age 49.6 (SD 4.2) 82% female	To understand the illness experiences of middle-aged adults with early knee OA.	Qualitative Semi structured interviews
Long[41] 2016 Netherlands	Not defined	172 participants	Patients recruited from orthopaedic outpatient offices	Mean 65 (SD 11) 67% female	To identify the needs of patients and physicians when deciding about treatment of hip or knee OA	Quantitative Questionnaires
Mann[42] 2011 UK	Clinically diagnosed lower-extremity OA	16 patients	38 patients contacted (purposive sampling) from a GP clinic	Age range 56-81yrs 56% female	To explore the opinions of patients and health professionals about the provision of health care for people with OA and possible service improvements	Qualitative Focus groups for patients
Mora[43] 2012 USA	Self-reported OA.	4478 participants	Data were obtained from an online patient educational program	Age range 29–100 years 62% female	To examine if a gender difference can be identified in the frequency and types of questions submitted by patients scheduled for total hip or total knee arthroplasty	Quantitative Web based survey
Parsons[44] 2009 UK	Radiologically advanced OA of the hip or knee	6 participants	Purposive sampling of patients of preoperative assessment unit with had advanced OA of the hip or knee, awaiting joint replacement.	Age range 60–76 years 50% female	To explore the lived experiences of patients with severe OA of the hip or knee while awaiting joint replacement surgery.	Qualitative Phenomenology, unstructured interviews
Pellinen[45] 2016 Finland	Not defined	252	Purposive sample of patients with knee OA recruited from health care centers	Mean age 68 (range 25–89) Gender unknown	To assess the socio-demographic and disease-related symptoms and emotions as well as the knowledge expectations of recently diagnosed patients with knee OA.	Quantitative Questionnaires
Rosemann[46] 2006 Germany	ICD codes of coxarthrosis or gonarthrosis.	20 participants	Patients randomly selected from GPs’ computer files by search for patients ICD codes of coxarthrosis or gonarthrosis.	Average age 56 (range 40–78 years) 60% female	To identify health care needs of patients with OA and to reveal possible obstacles for improvements in primary care management of OA patients.	Qualitative Semi-structured Interviews
Saroop-D’Souza[47] 2001 UK	Self reported OA	50 participants, 12 with OA	Convenience sampling, patients from an orthopaedic outpatient clinic	Mean age 46.8 (SD 17.04) 62% females	To establish the usefulness of an informational videotape on OA as perceived by patients and to explore the extent to which the outpatient department environment affect patients’ viewing of the tape.	Quantitative Questionnaires
Stark[48] 2014 Finland, Iceland, Sweden	Clinical diagnosis of OA	320 patients out of 445 participated	320 patients with OA on the waiting list for an elective hip replacement at one of 7 different hospitals	Mean age 64 (SD 11) 55% female	To describe the differences between received and expected knowledge in patients undergoing elective hip replacement in 3 Nordic countries and to analyse how these differences are related to patients’ characteristics, preoperative symptoms and emotions.	Quantitative Questionnaires
Victor[49] 2002 UK	Radiographic diagnosis of knee OA	170 participants	Patients recruited from general practices that have referred patients to the Rheumatology Department at St George’s Hospital	Average age 63 (range 45–90) 73% female	To explore the patients’ perspective on the meaning and significance of living with arthritis.	Qualitative Interviews and patient diaries
Washington[50] 2015 UK	Clinical diagnosis of OA	12 patients	8 clinicians working as advanced musculoskeletal practitioners were asked to invite all patients	Not described	To gain a perspective of patients’ experience of an online patient decision aid for osteoarthritis of the knee as a method of shared decision making in a Musculoskeletal Clinical Assessment and Treatment service	Mixed-methods Questionnaires
Willis[51] 2014 USA	Definition not specified	5 patients from each online arthritis-related communities	4 online arthritis-related communities were identified through popular search engines.	Age range 21–82 years 75% female	To understand the development of health literacy regarding chronic disease self-management by means of online health communities and the communication exchanged therein.	Qualitative Ethnomethodology

ACR:American College of Rheumatology, TKA:total knee arthroplasty, OA:osteoarthritis, RA:rheumatoid arthritis, PsA:psoriatic arthritis, SD:standard deviation, KL:Kellegren-Lawrence, ICD:International Classification of Diseases, ENAT:Educational Needs Assessment Tool, OENAT:Austrian-German Education Needs Assessment Tool

Participants were classified as having OA using American College of Rheumatology (ACR) criteria in 3 studies[29, 32, 34], radiographic change and pain in 4 studies[30, 40, 44, 49], self report in 6 studies[24, 27, 35, 39, 43, 47], chart review in 3 studies[31, 33, 46], clinical diagnosis in 4 studies[36, 37, 42, 48, 50], and by undefined methods in 8 studies[22, 23, 26, 28, 38, 41, 45, 51].

There were 16 qualitative studies[22–26, 28, 29, 31, 35, 36, 38, 42, 44, 46, 49–51]; 10 used semi-structured interviews[23, 27–31, 36, 40, 46, 49], 7 focus groups[23, 26, 34–36, 38, 42], 3 in depth interviews[24, 39, 44], 2 used diaries[39, 49] and 1 used ethnography [51]. The 11 quantitative studies used questionnaires[30, 32–34, 39, 41, 43, 45, 47, 48] and surveys [37]. Three studies used mixed methods[30, 39, 50].

While the size of study populations ranged from 5 to 4478 participants, most study populations were of modest size, with 22 studies having less than 100 participants[22–29, 31, 32, 34–36, 38–40, 42, 44, 46, 47, 50, 51]. Eight studies had more than 100 participants[37, 41] [30, 33, 43, 45, 48, 49]. The included studies had a female predominance, with 2 studies including only females[23, 24] and 22 studies having more females than males[22, 26, 28–37, 40–43, 47–49, 51, 52]. The mean age of participants in the included studies was 62 with an age range from 21–100 years of age.

### Quality of studies

Quality assessments of the included studies are presented as supplementary documents, (S1 and S2 Figs). The overall quality of qualitative studies was poor, especially for CASP criteria 4 to 6, reflecting potential biases with recruitment strategy and data collection (S1 Fig). The quantitative studies were of low quality: 10 studies were at high risk of bias[30, 32, 33, 37, 39, 41, 43, 45, 47, 48, 50] and 1 study was at moderate risk of bias[34] (S2 Fig).

### Results of review

Three main areas of patient perceived health information needs for OA emerged from the included studies.

#### Patients’ perceived need for clear communication of health information (Table 2)

**Table 2:** Patient perceived need for clear communication of health information.

Author & Year	Results
***Need for clear explanations***	
Alami 2011[22]	Patients were dissatisfied with the amount of knowledge received and unclear explanations
Barker 2014[25]	Patients wanted clear, easy to understand information
Baumann 2007[26]	Patients thought that practitioners were frequently not explicit enough when discussing the seriousness of the diagnosis or the value of certain drugs compared to others Patients wanted practitioners to use language they can understand.
Bayliss 2009[27]	Participants wanted clear communication of individualised care plans
***Words used in OA***	
Barker 2014[25]	Many terms used in OA are misunderstood by patients, such as “rheumatism”, “cartilage”, “rehabilitation” and “inflammation”. “Wear and tear” was considered approachable and easy to understand. However, there was a mixed response with women tending to respond negatively to the implication that ‘wear and tear’ was a sign of getting old and some patients associated it with a negative connotation that arthritis is untreatable or their GP is not taking their condition seriously. Only 1 participant could define the word effusion. Participants guessed that it meant “fusing” such as bones fusing together. Patients did not want the term effusion associated with their arthritis.
Jinks 2007[39]	Patients felt that a lack of effectiveness of treatments was reinforced when knee pain was linked to ageing, and particularly when the notion of “wear and tear” was mentioned in consultations Many participants felt that the concept of “wear and tear” have a negative impact on the thinking of health professionals, and in turn their patients.
***Communication style***	
Baumann 2007[26]	Patients reported that inappropriate gestures generate anxiety Silence from the practitioner was interpreted as “powerlessness”

Four studies reported that patients were dissatisfied with the unclear explanations provided by healthcare providers regarding their OA[22, 25–27]. Barker found that many terms used in OA were misunderstood by patients or had different connotations, such as “rheumatism”, “inflammation”, “cartilage” and “rehabilitation”[25]. Moreover, patients reported negative connotations with a number of words and phrases. For example, the term “effusion” was perceived to mean “fusion of bones” and patients did not want this associated with their arthritis. Furthermore, Jinks found that patients perceived “wear and tear” as being linked to ageing and reinforced a lack of effectiveness of treatments[39]. Four studies[22, 25–27] reported patients’ preferences with communication style, and found patients desired clear communication of individualised care plans from their health care providers[25–27]. Patients also reported that inappropriate gestures generated anxiety and that silence from the practitioner was interpreted as the doctors’ “powerlessness”[26].

#### Patients’ perceived need to obtain health information from a variety of sources (Table 3)

**Table 3:** Patient perceived need to obtain health information from a variety of sources.

Author & Year	Results
***Information provided by health professionals***	
Al-Taiar 2013[23]	Some patients noted a difference between private and public sector doctors in the way they provide information and explanations. Patients reported that public sector clinicians simply ask “do you want the surgery or not” and do not provide any written or verbal information about the surgery. Participants expressed full trust in their surgeons but at the same time expressed a strong sense of dissatisfaction with the insufficient amount of information provided.
Baird 2003[24]	Patients also seek information related to self-care from nurses, physicians and other health professionals in clinics and physician offices. Women said they obtain the most useful information from the nurse practitioner from the senior citizen centre.
Baumann 2007[26]	Advice and response to questions in particular about topics in the media were perceived as generally good Patients felt they had to ask for health care advice, rather than be given the information spontaneously
Brembo 2016[28]	Patients visit their GP to get an explanation of their pain, however, they felt that time provided by the clinician was a barrier. Patients perceived that they did not receive general information about OA and pain management from their GP.
Bayliss 2009[27]	Patients wanted more convenient access to providers via telephone or internet, as well as in person
Chan 2011[29]	Patients learned coping strategies from health professionals, the media, Internet, physical therapists, doctors and fellow sufferers.
Hill 2011[36]	Patients were dissatisfied with the perceived lack of understanding, the type of help and information received from some health care practitioners. They felt there was a contradiction in the advice and information given to some participants by various health care practitioners, which may indicate a lack of knowledge from the practitioner
Hofstede 2016[37]	Patients wanted sufficient time with the healthcare practitioner to explain everything
Ilic 2005[38]	Patients typically relied on their doctor for general medical information, but once diagnosed with OA, all participants stated they were keen to use the Internet as an alternative source of information
Mann 2011[42]	Patients expressed a desire to access someone with specialist knowledge of arthritis, possible a practice nurse who was easily accessible and knowledgeable
Parsons 2009[44]	Patients reported a lack of healthcare professional-led education/information sessions.
Roseman 2006[46]	Patients regarded specialists as an additional source of information Most patients stated they mostly trusted the information given by their GP about medications. To receive information and advice from practice nurses [printed information or lectures) was acceptable for most patients.
***Information provided by the Internet***	
Chan 2011[29]	Patients learned coping strategies from health professionals, the media, Internet, physical therapists, doctors and fellow sufferers.
Fedutes 2004[33]	Over half of the participants [57%) expressed interest in using an arthritis website. The patients that were not interested in a website gave reasons such as the physician or pharmacist answering all their questions, a lack of time and an absence of questions. Most interest was from patients less than 56 years of age and those with routine use of the internet
Grime 2014[35]	Not all of the patients read the summary and some found the OA guidebook difficult to read
Ilic 2005[38]	92% of participants stated that despite the use of a variety of search engines, sourcing relevant and credible health information from the Internet was difficult. Internet perceived as a source to obtain further information on the condition and potential treatments to supplement the information provided by their doctor by all. However, only 33% of participants eventually used the Internet to search for further information. The convenience of accessing medical information was a benefit of online information. Participants thought the reliability and credibility of the online information was variable, often requiring further investigation by cross-referencing other websites. Patients though the Internet User Guide enabled them to search and identify more relevant and scientific websites.
Long 2016[41]	Only 38% of patients felt that the Internet is a good way of delivery information
Washington 2016[50]	The use of an online patient decision aid gave patients a better understanding of OA than they gained from a discussion with a clinician
Willis 2014[51]	Patients use health communities on the Internet to seek information and share their experiences with others.
***Information provided by social media*, *print material***	
Baird 2003[24]	Participants purposefully seek information about arthritis and their health status through print media, experts at classes or on television, by consulting nurses and by listening to friends.
Chan 2011[29]	Patients learned coping strategies from health professionals, the media, Internet, physical therapists, doctors and fellow sufferers.
Cuperus 2013[31]	Some patients felt they had insufficient knowledge about OA and therefore read or used the information booklet. In relation to provision of an information booklet, some patients believed that the booklet is not a useful tool and did not read or use the booklet. The patients’ perception that OA is untreatable was a barrier to the use of the information booklet. Some patients were not willing to use an information booklet, as they believed they knew the information found in the booklet, they did not want to know everything about OA, they did not pay attention to their OA or felt to be sufficiently supported by their health care providers.
Long 2016[41]	Patients [68%) found booklets most suitable for delivering information
Saroop-D’Souza 2001[47]	In relation to information provision via video recordings, 80% of patients found the video useful but only 48% found it relevant. Two thirds of patients gained new information from the videotape.
***Information provided by support groups*, *family and friends***	
Al-Taiar 2013[23]	People who have had a total knee replacement were a source of information for patients considering an operation.
Baird 2003[24]	Participants purposefully seek information about arthritis and their health status through print media, experts at classes or on television, by consulting nurses and by listening to friends.
Brembo 2016[28]	Most participants found their social network of family and friends to be an invaluable source of information regarding joint replacement surgery. Patients felt that learning from others’ experiences provided hope for a better future. Those on the waiting list for joint replacement surgery felt well information about the operation, but they wanted more information about ways to prevent post-operative complications
Chan 2011[29]	Patients learned coping strategies from health professionals, the media, Internet, physical therapists, doctors and fellow sufferers.
Hofstede 2016[37]	Information and advice from friends and family were valued and facilitated non-surgical treatment options for OA
Parsons 2009[44]	Patients who knew someone who had undergone similar procedures considered themselves at an advantage in being able to share their experience. Support from friends, family and significant others who had undergone similar procedures were regarded as invaluable.

Information provided by health professionals. Twelve studies described patients’ utilisation of health professionals for information[23, 24, 26–29, 36–38, 42, 44, 46]. Patients sought health information from professionals with specialist knowledge of arthritis, such as physicians, other healthcare providers in clinics and nurse practitioners[24, 42, 46]. Some patients generally thought the advice and response to questions from their healthcare providers were good[26], however, they wanted more convenient access to healthcare practitioners[27]. Conversely, some patients were dissatisfied with the perceived lack of understanding and the type of information received from some healthcare practitioners[23, 36]. Also, patients have reported receiving contradictory information and advice from different healthcare providers, which was perceived as a lack of knowledge [36]. Patients wanted more time with the healthcare provider[28, 37] or more information sessions to provide further guidance and support[44]. They also wanted healthcare providers to be more forthcoming in giving health information[26]. Furthermore, some patients noted a difference in information provision between private and public sector doctors[23].

Information obtained from the Internet. Patients’ use of the Internet for health information was examined in 7 studies [29, 33, 38, 42, 51]. Patients used the Internet as a source of information [26, 38, 51] and to share their experiences with others [51]. In particular, patients accessed the Internet when information needs were not met by other sources [33, 42]. Most interest with using the Internet was from patients less than 56 years of age and those with routine Internet use [33]. However, Ilic found that although accessing medical information from the Internet was convenient, patients were concerned about the credibility and reliability of online information [38].

Information from other media including print and television. Patients’ use of other media, including printed materials, television and video recordings, for health information was evaluated in 6 studies[24, 29, 31, 35, 41, 47]. Baird reported that patients sought information about arthritis through print media or television[24], particularly if they felt they had insufficient knowledge about OA[31]. Cuperus[31] and Grime[35] found that some patients perceived potential from information booklets and read them, while Long reported that many patients found booklets most suitable for delivering information[41]. Furthermore, Saroop-D’Souza found that 80% of participants found an information video useful but only 48% found it relevant[47].

Information provided support groups, family and friends. Patients’ use of support groups, family and friends for health information was identified in six studies[23, 24, 28, 29, 37, 44]. Patients considering operative management sought information from other people who previously had a total knee replacement, and that learning from others’ experiences provided them with hope[23, 28, 44]. Patients also sought information about arthritis from classes or listening to friends[24]. Hofstede found that advice from family and friends facilitated non-surgical treatment options for patients with OA[37].

Gaps in the source of information. Two studies reported on patient perceived gaps in the health information sources[27, 40]. Kao found that patients did not know where to find information about OA and that there were few informative tools to help patients understand their disease[40]. Bayliss reported that patients preferred written information to aid understanding and recollection of information[27].

#### Patients’ perceived needs of health information content (Table 4)

**Table 4:** Patient perceived needs of health information content.

Author & Year	Results
***Demographic differences in content requirements***	
Dragoi 2013[32]	Female patients had significantly higher informational needs in most domains
Mora 2012[43]	Women asked more questions overall and they asked more questions about their condition, operative management and the risks and benefits of surgery.
Stark 2014[48]	Patients with higher education had more unfulfilled knowledge expectations Emotions such as fear, depressive state, concern and anxiety were related to unfulfilled knowledge expectations and depressive state was the major predictor of the variance in the difference between received and expected knowledge.
***Gaps in information provision—diagnosis***	
Baumann 2007[26]	Patients wanted information about the origins of disease Patients thought that practitioners were frequently not explicit enough when discussing the seriousness of the diagnosis or the value of certain drugs compared to others
Dragoi 2013[32]	A high percentage of patients expressed interest in receiving education about their arthritis.
Mann 2011[42]	Most patients expressed a strong desire for improved information about OA
Rosemann 2006[46]	Patients felt well informed about the cause and pathomorphology of disease. There was no request for more information about diagnostic aspects of OA. Patients thought that information on side effects was not that important to them because they were aware that many of the side effects mentioned on the package insert never occur. The majority of patients felt their GP tried to motivate them and explained the general effects of lack of exercise and being overweight.
Stark 2014[48]	Patients’ knowledge expectations were most fulfilled about symptoms related to the illness
***Gaps in information provision—prognosis***	
Baumann 2007[26]	Patients require more information about the prognosis and outlook of OA Patients wanted more information to help them accept the diagnosis and the uncertainty and doubt about the future
Mann 2011[42]	Most patients expressed a strong desire for improved information the likely progression of OA, especially at diagnosis and in the early stages of OA
***Gaps in information provision—management and prevention***	
Al-Taiar 2013[23]	Patients felt that medical advice to undertake total knee arthroplasty [TKA) came very late. Patients felt that a lack of information about TKA and this led to longer delays in undergoing surgery Some patients noted a difference between private and public sector doctors in the way they provide information and explanations. Patients reported that public sector clinicians simply ask “do you want the surgery or not” and do not provide any written or verbal information about the surgery. Participants expressed full trust in their surgeons but at the same time expressed a strong sense of dissatisfaction with the insufficient amount of information provided.
Baumann 2007[26]	Patients thought that information about recent developments in OA was inadequate Patients thought that practitioners were frequently not explicit enough when discussing the value of certain drugs compared to others Patients require more information in order to cope better with daily life and possible side effects of treatment Patients wanted more information as they feel that knowledge helps them communicate with practitioners and become partners in the management of OA Patients wanted information regarding prevention of OA in their children and grandchildren
Brembo 2016[28]	Those on the waiting list for joint replacement surgery felt well information about the operation, but they wanted more information about ways to prevent post-operative complications
Clarke 2014[30]	Patients’ dissatisfaction stem from limited information provided by doctors in terms of management options
Grime 2014[35]	Patients wanted more information in the guidebook about what they can do about OA, rather than be given a lot of medication detail about OA Patients wanted to know what people with OA can do for themselves. Patients wanted more information about the emotional impact of OA.
Hill 2011[36]	Patients were unsure about exercising their hands and fingers. Patients felt they should be given more information about the medication prescribed for them in order to make informed decisions about their treatment Patients emphasised the lack of information on assistive devices and some patients viewed this as a lack of recognition of the patients’ function problems related to hand OA
Hofstede 2016[37]	Patients believe that lifestyle advice was important and facilitated use of non-surgical treatments
Jinks 2007[39]	There is limited amount of discussion between GPs and other health professionals about the pros and cons of taking NSAIDS for knee pain, and patients in turn tended to make their own decisions about dosage
Long 2016[41]	Patients felt that a lack of information was the most important factor in making a decision about surgical treatment
Mann 2011[42]	Patients wanted information about diet and exercise, how to minimise OA symptoms and progression and practical information about aids and local services Some patients were not aware of other services such as occupational therapy Patients felt they lacked information to help them judge when to have a joint replacement
Mora 2012[43]	Regarding joint replacement surgery, the most common type of question asked was in the category of “Risks and Benefits”, followed by “Your Procedure” category.
Pellinen 2016[45]	The highest knowledge expectations were regarding pain management and care, prevention of joint injuries and exercise. The lowest knowledge expectations were regarding weight loss strategies.
Rosemann 2006[46]	Patients welcomed basic information on self-help groups, but they were unsure about potential benefits.
Stark 2014[48]	Patients felt that had limited information about financial support
Willis 2014[51]	Patients seek information about how to better manage their arthritis
***Gaps in information provision—source of information***	
Kao 2014[40]	Patients did not know where to find information about OA and there were few instructional tools to help patients understand OA
Bayliss 2009[27]	Patients wanted information in writing to aid understanding and to help patients remember

Demographic variations in content requirements. Four studies explored the demographic variation in OA health information needs[32, 43, 45, 48]. Dragoi[32] and Mora[43] evaluated the gender differences in health information needs and found that females had higher educational needs[32]. In particular, women asked more questions about their condition, operative management options and the risks and benefits of surgery[43, 45]. Stark reported that patients with higher education and those with depression or anxiety who were awaiting hip joint replacement surgery had more unfulfilled knowledge expectations and wanted more information[48].

Gaps in information about diagnosis. Five studies examined patients’ perceived gaps regarding diagnostic information[26, 32, 42, 46, 48]. There were conflicting data about the satisfaction with the amount of information provided, with some patients feeling well informed about the cause, symptomatology and pathomorphology of OA[46, 48], whilst other patients wanted more information about the origins of disease and more explicit details about the seriousness of the diagnosis[26]. Dragoi and Mann reported that patients expressed interest in receiving education about their arthritis[32, 42]. Some patients wanted more information about prevention of OA in their offspring[26].

Gaps in information about management options. Fourteen studies explored patients’ perceived gaps in OA management information[23, 26, 28, 30, 35–37, 39, 42, 43, 45, 46, 48, 51]. Clarke reported that patients were dissatisfied with the amount of information provided from medical practitioners about management options[30]. Patients wanted information about management strategies for OA, particularly about medications[26, 36], assistive devices[36], diet and weight management[37, 42], exercise therapy and occupational therapy[36, 37, 42], symptom control[42, 45] and self-management strategies[35, 51]. They also wanted information regarding local services[36], support groups[46] and financial support[45, 48]. Moreover, patients felt that they lacked information about surgical options[23, 41, 42], especially details about joint replacement surgery[23, 28, 43], and if provided, patients felt that medical advice about arthroplasty came very late[23]. Patients also wanted more information about recent developments in the management of OA[26]. Brosseau and Mann found that patients required more OA management information to help them cope with daily life and self-manage their OA[26, 42]. Patients reported that more information enabled improved communication with health care practitioners, which empowered them to be more involved in the management of their disease[26].

Gaps in information about prognosis. Patient perceived gaps in prognostic information was evaluated in 2 studies[26, 42]. Baumann found that patients required more information about the prognosis and progression of OA[26]. In particular, Mann reported that patients desired prognostic information at the time of diagnosis or early stages of disease[42].

## Discussion

To improve the uptake of OA clinical practice guideline recommendations by patients and to support co-care and promote patient self-management, the mismatch between patients’ perceived health information needs and the current delivery of information needs to be better aligned. In this scoping review, we identified a number of areas of patients’ perceived need for health information: (1)the need for clear communication of information, (2)the need to obtain information from a variety of sources and (3)the content needs of health information.

Patients consistently desired information to be delivered using clear and simple language, presented in a positive and constructive manner[22, 25, 26]. However, the language used by healthcare providers to convey health information is frequently misinterpreted by patients[25], and associated with negative connotations[25, 39]. These findings are similar to previous research evaluating medical terms used in back pain care[53]. Thus, in supporting patients with OA, healthcare professionals and information providers cannot assume patients’ comprehension or that the terms used are acceptable to patients. Given the misunderstandings and potential problems related to the use of words, such as “degenerative change”, future initiatives to develop information tools for OA should include patients. This may allow alignment of the language used to the appropriate level and improve the healthcare provider-patient relationship, enabling better patient engagement in the active management of OA. Whilst there are recent international consensus recommendations about what patients with hip and knee OA need to know, these guidelines have been determined largely by clinician researchers and may not have sufficiently incorporated the patient perspective to optimise patient engagement: further research is required[54].

Patients obtain health information from a variety of sources[23, 24, 27–29, 31, 33, 35, 38, 42, 44, 46, 47, 51]. This review found that patients sought information about OA from healthcare providers with specialist knowledge of arthritis[24, 27, 29, 38, 42], despite voicing concerns about the difficulties with access to medical practitioners[27] and the quality of information provided[23, 36]. Medical practitioners have identified a lack of availability of quality resources and time restraints as a major barrier to providing OA health information[42, 55]. Given that patients are receptive to receiving information from other healthcare providers including nurse practitioners[24, 42, 46], other avenues of health information from services allied to medicine may be utilised. This can provide more convenient and cost-effective access to health information, which has also been used in other conditions such as rheumatoid arthritis[56, 57].

Other sources of information that patients utilise include print media and television[24, 29, 31, 41, 47], support groups or from family and friends[23, 24, 28, 29, 37, 44]. Thus, it is clear that patients obtain information from a variety of sources, both healthcare related and lay sources. This may be due to dissatisfaction with the amount and content of information provided from an individual source, or it may be that patients want information from complementary sources to provide a more individualised and holistic approach to their disease. In particular, patients perceived the use of lay sources for information regarding operative management to be invaluable[23, 44], as patients sought information from people with previous experience, and this information was deemed more relatable and credible. Optimising information provision from a variety of complementary sources may improve patient understanding of the condition and enable more efficient information delivery, reducing dependence on primary healthcare doctors.

Furthermore, patients expressed interest in using the Internet to obtain information, particularly when they have otherwise unmet health information needs[29, 33, 38]. However, they have concerns about the credibility of information[38]. It is possible that as the use of technology becomes more widespread, and computer literate individuals age, the use of the Internet as a source of information will increase. Patients desire empowerment and are keen to be actively involved in their own health; therefore they seek different methods of health information delivery to address a diverse range of perceived needs. Thus, the use of online communities is becoming more common[58, 59], and provides an avenue for patients to obtain health information that is accessible and allows patients with similar experiences to share self-management strategies and advice that is holistic and individualised[51, 59–61]. It also provides social support and interaction between patients with similar shared experiences[51, 58, 59, 62]. There is emerging evidence that Web-based resources providing health information to patients with OA have improved the quality of life of its users and supported self-management[63]. Further research is required to explore and integrate the role of developing technologies in the provision of more effective and efficient health information, as despite the availability of the internet, information content needs persist.

Several studies in this review explored the perceived needs of health information content. These identified demographic differences in the perceived health information needs of patients with OA. Females had consistently higher health information needs than males[32, 43, 45]. Whilst this finding may be due to sampling bias with a predominance of female participants in the included studies, this is congruent with other studies evaluating patients with a variety of arthritides[32, 64]. These studies have demonstrated that women show more interest in disease management than men, and that men with arthritis prioritise work commitments over health concerns which may affect their perceived health information needs[32, 64]. Furthermore, those with higher education[48] had more unfilled health information expectations. The review included studies over a wide timeframe—some 26 years. As there was limited data, we were limited in our ability to examine changes in health information needs over time as a primary aim of the review and in a systematic manner. Nevertheless, the available data suggest that the nature of health information needs did not change over the study period. Surprisingly, none of the included papers identified the role of digital technologies in delivery of health information, although it would be reasonable to expect that an evidence in this area will emerge in time. There is limited data examining the influence of other variables, such as socioeconomic status and medical co-morbidities, on the health information needs of patients with OA. Further studies are needed to assess whether addressing the health information needs of subgroups that desire more information translates into improved health outcomes in OA. Health information needs tend to differ among people with back pain according to level of disability[65], so it is likely that the same issue applies to people with OA, although primary research should be undertaken to confirm this hypothesis.”

Patients have also identified specific gaps in the provision of health information. Despite the current recommendations for the provision of health information to patients with OA[11, 54], patients have reported dissatisfaction with amount and clarification of knowledge[22, 23, 26, 36, 48, 49], particularly about management options, the prognosis of OA and prevention of worsening OA[26, 28, 30, 36, 39, 41, 44, 45]. In particular, patients have observed an apparent paucity of information about assistive devices and exercise therapy, which is perceived as a lack of recognition of the functional limitations of the disease by healthcare providers[36]. This underscores the misalignment in the perceived needs between healthcare providers and patients[66, 67]. Healthcare practitioners tend to underestimate the impact or severity of patients’ symptoms, and prioritise management options differently to patients [22, 42, 66–68]. This may be a reflection of the limitations of healthcare providers and their lack of knowledge of the benefits of non-pharmacological and non-surgical care options for OA [69, 70]. Consequently, the priorities and perceived needs of patients may not be addressed, thus impeding on the patient’s adherence to treatment recommendations and their willingness to actively participate in their own care.

There are a number of limitations to this review. Firstly, despite utilising a comprehensive and inclusive search strategy, only 30 studies were identified to be relevant for this scoping review. This highlights an urgent need for future research initiatives to examine patients’ perceived needs for health information for osteoarthritis. Also, as few studies directly examined the patients’ perspective of their health information needs regarding OA, the categories of need emerging were extrapolated from heterogeneous studies evaluating different study questions with varied populations. Most of the included studies have small sample sizes, consist mainly of women with hip or knee OA and were conducted in developed, English-speaking countries, mainly the United Kingdom. Additionally, the majority of studies recruited participants from hospital settings or general practices, rather than community centres. Therefore, the study populations may not be representative of all community dwellers with OA or transferable to people in low and middle-income economies, which may limit the generalizability of the results. Research addressing consumers’ health information needs related to OA in the context of low and middle income settings and with consideration to community dwelling individuals should be prioritised and would support complementary efforts undertaken by the World Health Organisation in this area. Some of the included studies are over 10 years old, and may not reflect current patient health information needs. Furthermore, many of the included studies were susceptible to bias, and thus were of limited quality. However, as this was a scoping review, the main concern relates to a failure to capture populations that were not included and needs that were not addressed in any study directly. This review has been focussed on identifying patients’ perceived needs of health information and did not explore the effectiveness of communication, the availability of health information or the accuracy of patients’ knowledge of OA. Identifying these factors and where they deviate from patient perceived needs may improve health information delivery.

Despite these limitations, this review has provided a comprehensive summary of the existing literature from four complementary databases and incorporates both qualitative and quantitative studies to capture the breadth of the topic. By performing an inclusive scoping review, this has allowed a richer description of the patient experiences and perceived needs, spanning across all disciplines of OA health care than would have been possible otherwise. We have included all identified perceived needs, regardless of the quality of the evidence. This is necessary to capture to breadth of evidence available and to record all reported patients’ needs. However, we acknowledge that any finding in a single study requires validation in another study. Thus, while we have included all relevant literature, it is possible that some needs may not have been addressed within the existing literature, and as such, we cannot conclude that the evidence we have synthesised is exhaustive on the topic (eg the role of new digital technology in health information provision). This must be taken into consideration when accepting the conclusions.

**Fig 2:**
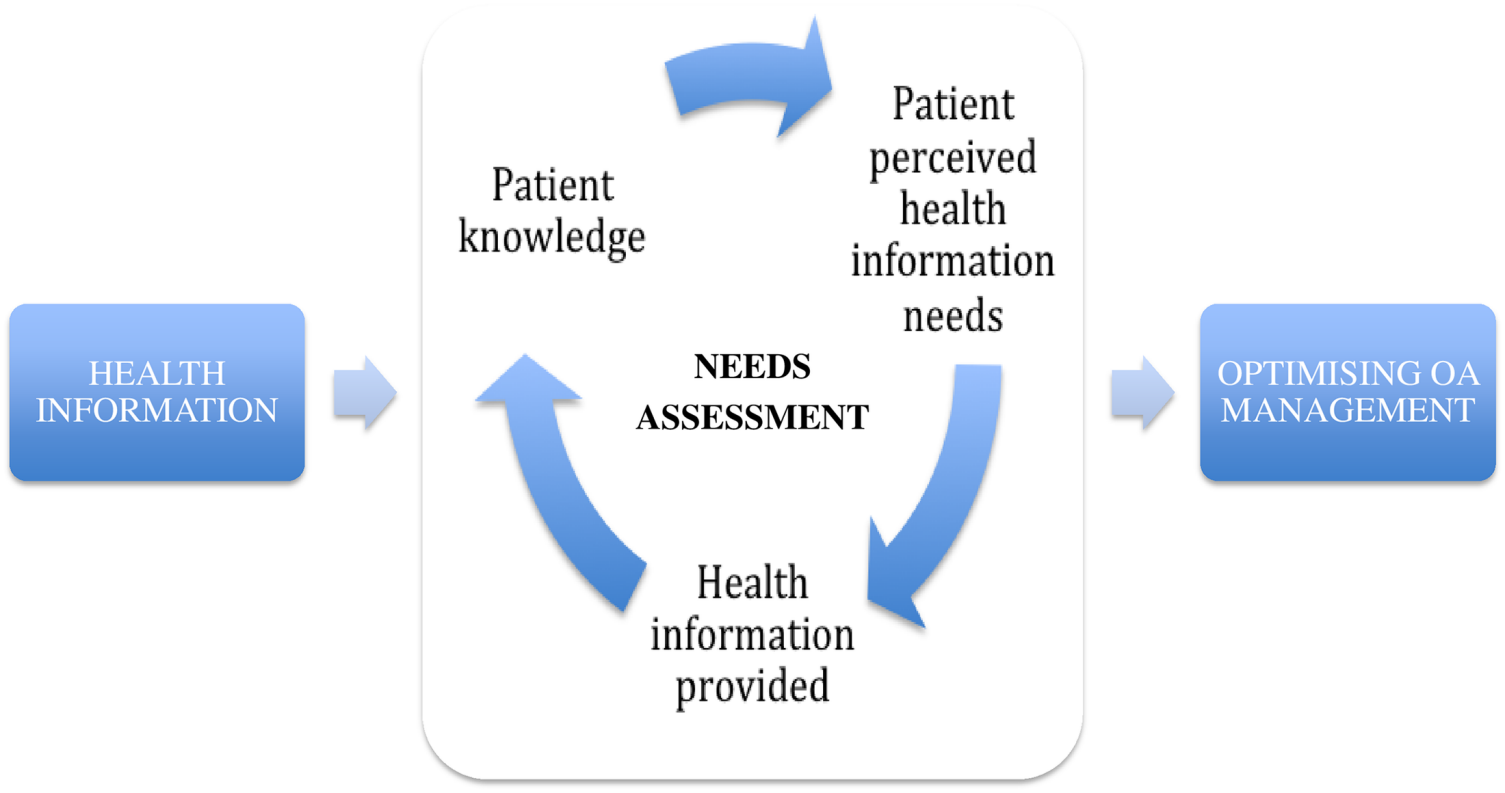
Conceptualising health information needs assessment.

To address whether the perceived needs of patients are a true reflection of need, a needs assessment is required. This involves a complex, iterative process of exploring patient health literacy, their perceived health information needs and an understanding of the content of health information provided and resource allocation (Fig 2). These results should also be taken in conjunction with patients’ perceived needs of health services, which are motivated by the need for symptom control and largely aligned with existing guidelines[71]. Our results suggest that there are gaps in current content and mode of delivery of OA health information. This may adversely impact the uptake of OA management guidelines and recommendations that require active patient participation, such as exercise therapy. The costs of healthcare are rising[72], yet at the same time, the available resources are limited. The results of this review will be useful to assist healthcare providers and policy-makers to better understand the perceived needs of patients, informing future management strategies and guidelines, taking into account the patient perspective. Moving forward, when implementing guidelines, healthcare providers may need to provide more individualised information to patients regarding the diagnosis and management of OA and utilise multiple modes of information delivery to provide patient-centred care and optimise patient uptake of their recommendations. Further education should also be provided to healthcare providers to equip them with the knowledge and skills required to manage patients with OA and also to enhance their communication skills to convey the appropriate messages. Moreover, patients should be involved in developing guidelines and patient information material, incorporating the patient perspective. This may improve the communication of health information to patients, using appropriate language that better aligns with their preferences and expectations[54]. There is a gap in the evidence about the effects of this partnership[73], which should be evaluated in future studies to assess whether patient involvement in developing patient information material ultimately translates into improved OA outcomes.

## Conclusions

We have used a broad systematic scoping review of the literature to identify patients’ health information needs relating to OA. We found that patients are dissatisfied with the delivery of health information, as well as the content provided, particularly regarding the management options, prognosis of OA and preventive strategies. This review helps to understand how patients’ needs relate to existing guidelines and where they deviate. Identifying these gaps will improve our ability to develop strategies to better align patients with evidence-based practice, promote more effective self-management and increase the uptake of recommendations from guidelines. To do this more successfully we can utilise novel information delivery strategies, using a variety of complementary sources of information. These may result in better health outcomes for patients with OA.

## Supporting information

S1 Fig Quality assessments of qualitative studies. (DOCX)

S2 Fig Quality assessments of quantitative studies. (DOCX)

S3 Fig PRISMA checklist. (DOC)

S1 Text Search strategy. (DOCX)

## Abbreviations

OA: osteoarthritis
