# Supplementary material for: Patients’ perceived needs of osteoarthritis health information: A systematic scoping review
Source: PLoS One. 2018 Apr 16;13(4):e0195489. doi: 10.1371/journal.pone.0195489 (PMC5901923; doi:10.1371/journal.pone.0195489)
Supplement: S1 Fig — (DOCX) [file pone.0195489.s001.docx]

| **STUDY** | | **CASP 1^1^** | | **CASP 2^2^** | | **CASP 3^3^** | **CASP 4^4^** | **CASP 5^5^** | **CASP 6^6^** | **CASP 7^7^** | **CASP 8^8^** | **CASP 9^9^** | **CASP 10^10^** |
| --- | --- | --- | --- | --- | --- | --- | --- | --- | --- | --- | --- | --- | --- |
| Al-Taiar ^22^ | |  | |  | |  |  |  |  |  |  |  |  |
| Alami ^21^ | |  | |  | |  |  |  |  |  |  |  |  |
| Baird ^23^ | |  | |  | |  |  |  |  |  |  |  |  |
| Barker ^24^ | |  | |  | |  |  |  |  |  |  |  |  |
| Baumann ^25^ | |  | |  | |  |  |  |  |  |  |  |  |
| Bayliss ^26^ | |  | |  | |  |  |  |  |  |  |  |  |
| Brembo^49^ | |  | |  | |  |  |  |  |  |  |  |  |
| Chan ^20^ | |  | |  | |  |  |  |  |  |  |  |  |
| Clarke ^27^ | |  | |  | |  |  |  |  |  |  |  |  |
| Cuperus ^28^ | |  | |  | |  |  |  |  |  |  |  |  |
| Grime ^32^ | |  | |  | |  |  |  |  |  |  |  |  |
| Hill ^55^ | |  | |  | |  |  |  |  |  |  |  |  |
| Ilic ^34^ | |  | |  | |  |  |  |  |  |  |  |  |
| Jinks ^35^ | |  | |  | |  |  |  |  |  |  |  |  |
| Kao ^36^ | |  | |  | |  |  |  |  |  |  |  |  |
| Mann ^37^ | |  | |  | |  |  |  |  |  |  |  |  |
| Parsons ^39^ | |  | |  | |  |  |  |  |  |  |  |  |
| Rosemann ^40^ | |  | |  | |  |  |  |  |  |  |  |  |
| Victor ^43^ | |  | |  | |  |  |  |  |  |  |  |  |
| Washington ^45^ | |  | |  | |  |  |  |  |  |  |  |  |
| Willis ^44^ | |  | |  | |  |  |  |  |  |  |  |  |
| Legend: |  | Yes |  | No |  | Can’t tell | | | | | | | |

**S1 Fig Quality assessment of qualitative studies**

^1^CASP 1: Was there a clear statement of the aims of the research

^2^CASP 2: Is a qualitative methodology appropriate?

^3^CASP 3: Was the research design appropriate to address the aims of the research?

^4^CASP 4: Was the recruitment strategy appropriate to the aims of the research?

^5^CASP 5: Was the data collected in a way that addressed the research issue?

^6^CASP 6: Has the relationship between researcher and participants been adequately considered?

^7^CASP 7: Have ethical issues been taken into consideration?

^8^CASP 8: Was the data analysis sufficiently rigorous?

^9^CASP 9: Is there a clear statement of findings?

^10^CASP 10: How valuable is the research?
