# Supplementary material for: Patients’ perceived needs of osteoarthritis health information: A systematic scoping review
Source: PLoS One. 2018 Apr 16;13(4):e0195489. doi: 10.1371/journal.pone.0195489 (PMC5901923; doi:10.1371/journal.pone.0195489)
Supplement: S2 Fig — (DOCX) [file pone.0195489.s002.docx]

**S2 Fig Quality assessments of quantitative studies**

| STUDY | | Criteria 1^1^ | Criteria 2^2^ | | Criteria 3^3^ | Criteria 4^4^ | Criteria 5^5^ | Criteria 6^6^ | Criteria 7^7^ | Criteria 8^8^ | Criteria 9^9^ | Criteria 10^10^ |
| --- | --- | --- | --- | --- | --- | --- | --- | --- | --- | --- | --- | --- |
| Clarke ^27^ | |  |  | |  |  |  |  |  |  |  |  |
| Dragoi ^29^ | |  |  | |  |  |  |  |  |  |  |  |
| Fedutes ^30^ | |  |  | |  |  |  |  |  |  |  |  |
| Gignac ^31^ | |  |  | |  |  |  |  |  |  |  |  |
| Hofstede ^47^ | |  |  | |  |  |  |  |  |  |  |  |
| Jinks ^35^ | |  |  | |  |  |  |  |  |  |  |  |
| Long ^46^ | |  |  | |  |  |  |  |  |  |  |  |
| Mora ^38^ | |  |  | |  |  |  |  |  |  |  |  |
| Pellinen^48^ | |  |  | |  |  |  |  |  |  |  |  |
| Saroop- D'Souza ^41^ | |  |  | |  |  |  |  |  |  |  |  |
| Stark ^42^ | |  |  | |  |  |  |  |  |  |  |  |
| Washington ^45^ | |  |  | |  |  |  |  |  |  |  |  |
| Legend: |  | Yes |  | No | | | | | | | | |

^1^Criteria 1: Was the study’s target population a close representation of the national population in relation to relevant variables?

^2^Criteria 2: Was the sampling frame a true or close representation of the target population?

^3^Criteria 3: Was some form of random selection used to select the sample OR was a census taken?

^4^Criteria 4: Was the likelihood of nonresponse bias minimal?

^5^Criteria 5: Were data collected directly from the subjects?

^6^Criteria 6: Was an acceptable case definition used in the study?

^7^Criteria 7: Was the study instrument that measured the parameter of interest shown to have validity and reliability?

^8^Criteria 8: Was the same mode of data collection used for all subjects?

^9^Criteria 9: Was the length of the shortest prevalence period for the parameter of interest appropriate?

^10^Criteria 10: Were the numerator(s) and denominator(s) for the parameter of interest appropriate?
