# Supplementary material for: Patients’ perceived needs of osteoarthritis health information: A systematic scoping review
Source: PLoS One. 2018 Apr 16;13(4):e0195489. doi: 10.1371/journal.pone.0195489 (PMC5901923; doi:10.1371/journal.pone.0195489)
Supplement: S1 Text — (DOCX) [file pone.0195489.s004.docx]

**S1 Search Strategy**

| 1. (consumer* or patient* or client* or customer* or service user*).tw. |  |
| --- | --- |

| 2. patients/ or inpatients/ or outpatients/ |  |
| --- | --- |

| 3. 1 or 2 |  |
| --- | --- |

| 4. (inform* or counsel* or understand* or communicat* or knowledge* or comprehen* or educat* or competen* or teach* or ability or capacity or capability or learn* or resource* or self efficacy or self management or self care or instruct* or decision* or train* or curricul* or message* or aware* or coach* or skill* or content or course* or program* or litera* or advi#e or resource* or source* or interven* or guid* or checklist* or news or research or topic* or form? or format*).tw. |  |
| --- | --- |

| 5. ((mass or communication or electronic or digital or multi or print* or visual or audiovisual or social or new or virtual or online or on line) adj (media or medium*)).tw. |  |
| --- | --- |

| 6. (internet or web* or online or on line or virtual* or electronic* or social network*).tw. |  |
| --- | --- |

| 7. (blog* or facebook or twitter or tweet* or instagram or youtube or google or podcast*).tw. |  |
| --- | --- |

| 8. ((video or online or on line) adj3 (game* or gaming)).tw. |  |
| --- | --- |

| 9. (screen? or ipad* or i pad* or tablet* or phone* or telephon* or iphone* or i phone* or smartphone* or ipod* or i pod* or mp3 player*).tw. |  |
| --- | --- |

| 10. (sms or instant messag* or text*).tw. |  |
| --- | --- |

| 11. ((electronic or handheld or digital or mobile) adj device*).tw. |  |
| --- | --- |

| 12. ((bill or notice or discussion or bulletin) adj board*).tw. |  |
| --- | --- |

| 13. (radio or television or tv or audio* or video* or tape* or recording* or cassette* or cd* or compact disc* or dvd* or motion picture* or film*).tw. |  |
| --- | --- |

| 14. (hotline* or answering service*).tw. |  |
| --- | --- |

| 15. (photo* or publication* or newsletter* or brochure* or booklet* or pamphlet* or leaflet* or flyer* or handout* or poster* or illustrat* or picture* or pictogram* or graphic* or icon* or image*).tw. |  |
| --- | --- |

| 16. (paper or print* or postal or mail* or letter* or correspondence or written or writing).tw. |  |
| --- | --- |

| 17. ((e or electronic) adj (mail* or prescrib* or health or learn*)).tw. |  |
| --- | --- |

| 18. ((automat* or interactive*) adj3 (telephon* or phone or voice or hotline or hot line)).tw. |  |
| --- | --- |

| 19. ((voice or speech) adj (response* or recognition or messag* or system* or technolog*)).tw. |  |
| --- | --- |

| 20. (telemedicine or telehealth or telecare or telemanag* or telenursing or telepharmac*).tw. |  |
| --- | --- |

| 21. (doctor* or physician* or practitioner* or health professional* or nurse* or allied health or physiotherapist* or physical therapist* or chiropractor* or occupational therapist*).tw. |  |
| --- | --- |

| 22. 4 or 5 or 6 or 7 or 8 or 9 or 10 or 11 or 12 or 13 or 14 or 15 or 16 or 17 or 18 or 19 or 20 or 21 |  |
| --- | --- |

| 23. (use* or using or utili* or need* or prefer* or interven* or service* or disseminat* or seek* or retriev* or transfer* or campaign* or provide* or provision or resource* or source* or aid* or promot* or access* or search* or find* or understand* or evaluat* or apprais* or assess* or demand* or relation* or insufficien* or deficit* or gap* or barrier* or enabler* or facilitat* or communicat* or deliver* or implement*).tw. |  |
| --- | --- |

| 24. ((consumer* or patient* or client* or customer*) adj4 (inform* or counsel* or understand* or communicat* or knowledge* or comprehen* or educat* or competen* or teach* or ability or capacity or capability or learn* or resource* or self efficacy or self management or self care or instruct* or decision* or train* or curricul* or message* or aware* or coach* or skill* or content or course* or program* or litera* or advi#e or resource* or source* or interven* or guid* or checklist* or news or research or topic* or form? or format* or ((mass or communication or electronic or digital or multi or print* or visual or audiovisual or social or new or virtual or online or on line) adj (media or medium*)) or (internet or web* or online or on line or virtual* or electronic* or social network*) or (blog* or facebook or twitter or tweet* or instagram or youtube or google or podcast*) or ((video or online or on line) adj3 (game* or gaming)) or (screen? or ipad* or i pad* or tablet* or phone* or telephon* or iphone* or i phone* or smartphone* or ipod* or i pod* or mp3 player*) or (sms or instant messag* or text*) or ((electronic or handheld or digital or mobile) adj device*) or ((bill or notice or discussion or bulletin) adj board*) or (radio or television or tv or audio* or video* or tape* or recording* or cassette* or cd* or compact disc* or dvd* or motion picture* or film*) or (hotline* or answering service*) or (photo* or publication* or newsletter* or brochure* or booklet* or pamphlet* or leaflet* or flyer* or handout* or poster* or illustrat* or picture* or pictogram* or graphic* or icon* or image*) or (paper or print* or postal or mail* or letter* or correspondence or written or writing) or ((e or electronic) adj (mail* or prescrib* or health or learn*)) or ((automat* or interactive*) adj3 (telephon* or phone or voice or hotline or hot line)) or ((voice or speech) adj (response* or recognition or messag* or system* or technolog*)) or (telemedicine or telehealth or telecare or telemanag* or telenursing or telepharmac*) or (doctor* or physician* or practitioner* or health professional* or nurse* or allied health or physiotherapist* or physical therapist* or chiropractor* or occupational therapist*)) adj4 (use* or using or utili* or need* or prefer* or interven* or service* or disseminat* or seek* or retriev* or transfer* or campaign* or provide* or provision or resource* or source* or aid* or promot* or access* or search* or find* or understand* or evaluat* or apprais* or assess* or demand* or relation* or insufficien* or deficit* or gap* or barrier* or enabler* or facilitat* or communicat* or deliver* or implement*)).tw. |  |
| --- | --- |

| 25. health education/ or consumer health information/ or patient education as topic/ or sex education/ |  |
| --- | --- |

| 26. needs assessment/ |  |
| --- | --- |

| 27. exp Health Promotion/ |  |
| --- | --- |

| 28. exp Attitude to Health/ |  |
| --- | --- |

| 29. self care/ |  |
| --- | --- |

| 30. communication barriers/ |  |
| --- | --- |

| 31. informed consent/ or truth disclosure/ |  |
| --- | --- |

| 32. health communication/ or exp information literacy/ or information seeking behavior/ |  |
| --- | --- |

| 33. decision making/ or choice behavior/ |  |
| --- | --- |

| 34. communications media/ or library materials/ or teaching materials/ or telecommunications/ or electronic mail/ or telemedicine/ or remote consultation/ or telephone/ or answering services/ or exp cellular phone/ or television/ |  |
| --- | --- |

| 35. exp Mass Media/ |  |
| --- | --- |

| 36. computers/ or exp Microcomputers/ or Minicomputers/ or exp Internet/ or electronic mail/ or video games/ |  |
| --- | --- |

| 37. exp Professional-Patient Relations/ |  |
| --- | --- |

| 38. 25 or 26 or 27 or 28 or 29 or 30 or 31 or 32 or 33 or 34 or 35 or 36 or 37 |  |
| --- | --- |

| 39. 2 and 38 |  |
| --- | --- |

| 40. 24 or 39 |  |
| --- | --- |

| 41. (need* or want* or like* or interest* or prefer* or satisf* or perspective* or experience* or attitude* or belief* or practice* or concern* or support* or participat* or advoca* or center* or centr* or orient* or focus* or empower* or expect* or opinion* or view* or perceive* or perception* or tailor* or bespoke or involv* or priorit*).tw. |  |
| --- | --- |

| 42. ((consumer* or patient* or client* or customer*) adj4 (need* or want* or like* or interest* or prefer* or satisf* or perspective* or experience* or attitude* or belief* or practice* or concern* or support* or participat* or advoca* or center* or centr* or orient* or focus* or empower* or expect* or opinion* or view* or perceive* or perception* or tailor* or bespoke or involv* or priorit*)).tw. |  |
| --- | --- |

| 43. exp Consumer Satisfaction/ or exp Consumer participation/ |  |
| --- | --- |

| 44. exp Perception/ |  |
| --- | --- |

| 45. 28 or 43 or 44 |  |
| --- | --- |

| 46. 42 or 45 |  |
| --- | --- |

| 47. 40 and 46 |  |
| --- | --- |

| 48. exp osteoarthritis/ |  |
| --- | --- |

| 49. (degen* adj4 arth*).tw. |  |
| --- | --- |

| 50. osteoarth*.tw. |  |
| --- | --- |

| 51. coxarth*.tw. |  |
| --- | --- |

| 52. gonarth*.tw. |  |
| --- | --- |

| 53. 48 or 49 or 50 or 51 or 52 |  |
| --- | --- |

| 54. 47 and 53 |
| --- |
